# Piloting a Peer Support Program for Patients Who Screen Positive for Intimate Partner Violence, Suicidal Ideation, and Depression

**DOI:** 10.3390/healthcare11172422

**Published:** 2023-08-30

**Authors:** Michelle Drouin, Mindy Flanagan, Jeanne Carroll, Connie Kerrigan, Heather Henry, Tammy Toscos

**Affiliations:** 1 Parkview Mirro Center for Research and Innovation, Fort Wayne, IN 46845, USA; mindy.flanagan@parkview.com (M.F.); jeanne.carroll@parkview.com (J.C.); tammy.toscos@parkview.com (T.T.); 2 Department of Psychology, Purdue University Fort Wayne, Fort Wayne, IN 46805, USA; 3 Parkview Behavioral Health Institute, Fort Wayne, IN 46805, USA; connie.kerrigan@parkview.com (C.K.); heather.henry@parkview.com (H.H.)

**Keywords:** intimate partner violence, suicidality, mental health, peer support, peer navigators, health systems, suicide prevention

## Abstract

Peer support programs have been utilized for a variety of mental-health-related needs, including suicidality and depression. In this pilot program, we developed a peer support network to address multimorbidity involving intimate partner violence (IPV), suicidal ideation, and depression. Over one year, our Suicide Obviation Support (SOS) navigators enrolled and provided at-elbow support to 108 patients (67.6% women) who screened positive for IPV, many of whom also screened at moderate or high risk for suicidality (64.8%) and/or exhibited depression symptoms. At a 6-month follow-up, 63 participants (58.3%) were retained. Those who stayed enrolled in the program for six months were less likely to report IPV and depression symptoms and were at a lower risk for suicide than the original sample, and analyses showed that IPV, depression, and suicide risk scores declined significantly in this group. The SOS navigators provided direct support and continuity of care for these high-risk patients, which included referrals to mental health treatment and other types of support services, such as transportation and emergency housing. This program provides a model for healthcare systems that desire to implement peer support programs servicing individuals who face multiple, acute mental health care needs.

## 1. Introduction

Health systems often face the challenge of treating patients who have multiple risk factors and co-occurring mental and/or physical health conditions. Treating multimorbid patients, defined as those having multiple chronic conditions, including mental health conditions, requires a holistic approach with special attention to continuity of care [1]. According to individuals who use mental health services, peer support is one critical component of this continuity of care [2]. Therefore, for this project, we piloted a peer support program for patients with multiple risk factors who presented to domestic violence (DV) shelters, emergency departments, and other mental health facilities affiliated with a midsized, Midwestern hospital. More specifically, we focused on a population of patients who screened positive for intimate partner violence (IPV), many of whom had attempted suicide and had depression+ symptoms.

According to CDC statistics, approximately 41% of women and 26% of men have experienced IPV in their lifetimes [3], and approximately 9 million women and men experienced IPV in the last year [4]. In Indiana, statistics are similar, with 43% of women and 28% of men reporting lifetime experience of IPV [5]. The Indiana Domestic Violence Counts 2022 report, which captures a count of domestic violence calls and services for one day across Indiana, found 1769 victims being served and 116 unmet needs for services, largely due to a lack of resources for emergency shelter [6].

Meanwhile, suicide is among the top nine causes of death for people ages 10 to 64 and the second leading cause of death for people ages 20 to 34 [7]. In 2021 alone, there were 1.7 million suicide attempts [7]. Rates of suicidality in the general population are somewhat similar among men and women: 0.5% of men and 0.6% of women report suicide attempts, and 4.1% of men and 4.5% of women report suicidal ideation [8]. Suicide risk has also been high throughout the Northeast Indiana counties in which our health system Emergency Departments (EDs) are located. According to electronic health records for 2011–2015, there were 413 suicide deaths, 1293 suicide-related hospitalizations, and 1971 suicide-related ED visits in these Northeast Indiana counties. This figure equates to approximately 700 suicide-related emergency department visits per year in our health system alone.

Decades of research shows that IPV may be a risk factor for suicide. In fact, a 2022 meta-analysis including 181 studies showed significant associations between IPV victimization and suicidal ideation among both women and men [9]. These results appear to replicate cross-culturally, as a recent analysis of World Health Organization data of women from 10 countries also shows an association between IPV and suicidality [10]. Notably, the rates for suicidal ideation among IPV victims are substantially higher than the current rates of suicidality in the general population. For example, in a recent study using a probability sample in England, rates for past-year suicidal thoughts among those who had ever experienced IPV were 29% among men and 51% among women [11]. These current statistics that include male victims of IPV are a notable contribution to the literature, as much of the previous research examining suicidality and IPV focused only on women victims of IPV. For example, a 1999 meta-analysis showed that among women IPV victims, the average prevalence rate of suicidality was approximately 18% [12], and a 2011 U.S. study in a large urban city showed 23% of women IPV victims had a history of suicide attempts [13].

Compared to male IPV victims, women victims tend to exhibit more negative mental health outcomes (e.g., anxiety and mood disorders), which includes a greater likelihood of suicidal ideation [9,11,14]. In fact, a previous systematic review showed that among women, IPV, suicide attempts, and depression were positively linked; however, among men, depression and IPV were associated in a negative direction, and there was no link between IPV and suicidality [15]. However, more recently, a South Korean study showed that past-year experiences of IPV were associated with suicidal ideation among both men and women [16]. There is also some evidence that IPV perpetration, as opposed to victimization, may be more associated with suicide among men. In a 2022 study of individuals who died by suicide and had close-in-time IPV-related events, 3% of men were IPV victims, 89% were perpetrators, and 1% were victims/perpetrators; whereas 27% of women were IPV victims, 51% were perpetrators, and 1% were victims/perpetrators [17]. Combined, these studies suggest that IPV and suicide are linked, though the associations between IPV victimization and suicidality among women appear to be stronger than those among men.

Domestic violence and mental health specialties have often worked independently of each other, which is a gap in care that needs to be addressed [18]. Although both suicidal patients and IPV victims were frequently seen in our EDs prior to the inception of this project, no suicide prevention or IPV services were provided through our standard of care. As part of a grant awarded to our hospital, an opportunity arose to develop a new program of action for addressing domestic violence and suicide in our community. Specifically, we scaled an existing peer support program to create a team of suicide obviation support (SOS) navigators to walk side by side in recovery with IPV victims and suicidal patients. Here, we provide a basic description of the program, as well as retention and mental health statistics (i.e., depression symptoms) for the patients involved, with the goal of providing a 6-month snapshot of patients involved in our SOS peer support program, as well as the main victories and challenges we faced during the program’s implementation. Our aim is to provide key findings for other health systems attempting to address multimorbidity of suicidality, IPV, and depression using a peer support program.

## 2. Materials and Methods

### 2.1. Peer Support Program

According to the Suicide Prevention Resource Center [19], a comprehensive approach to suicide prevention incorporates nine strategies, which include actions such as responding to crises and support through care transitions. This comprehensive approach provided the overarching structure for our program activities. As the previous approach to suicide prevention and care delivery to victims of DV in our community was fragmented across different systems and organizations, the key to our approach was to employ a network of Suicide Obviation and Support Navigators (SOS navigators) who were embedded in emergency departments (EDs) and connected (as support and training resources) to our inpatient psychiatric facility and local DV organizations and emergency shelters. SOS navigators provided at-elbow support and care coordination for patients discharged from the ED or inpatient facility and for patients obtaining services through organizations serving victims of DV. The goal of at-elbow support is to have an individual available 24 h a day to be a resource to the patient if they have questions or need help navigating the complexities of making appointments, coordinating medical care including behavioral health services, and accessing community support services (e.g., emergency housing) relevant to their specific needs. To address issues related to continuity of care, SOS navigators were assigned to patients within 24–48 h of referral, and all efforts were made for the same SOS navigator to assist the patient for the length of their involvement in the program.

#### 2.1.1. Program Background

The at-elbow navigator approach had precedence in our health system and had been utilized in the treatment of those who had recently experienced a drug overdose or were seeking opioid or substance use disorder (OUD/SUD) treatment. The peer navigators served as a critical referral source for treatment and other inpatient and outpatient OUD/SUD services, and they also engaged one-on-one with clients to help them navigate any aspects of recovery support with which the clients wished to engage (e.g., help with obtaining health care, food, or transportation). Hence, when we expanded our team to include SOS navigators, the extensive network was well-developed and integrative; the SOS navigators who covered the EDs had models to work collaboratively with peers based in other organizations (e.g., DV clinics, women’s and children’s hospital, inpatient treatment facilities, and a mobile integrated response system involving the local police department).

#### 2.1.2. Peer Support Program Components

During the course of this Parkview Health IRB-reviewed program evaluation (IRB record #PRC20–0928 SAMHSA S-DV) project, we hired, trained, and deployed six SOS navigators. These navigators were supervised by a nurse, and together they worked as part of a clinical team. Navigators typically met with clients in the EDs and other support facilities after a suicide attempt, report of suicidal ideation, and/or IPV-related events. There were several key aims of the program, designed to assist with continuity of care:

1. Crisis response: SOS navigators were to contact those referred to the program via text, calls, email, or mail within 48 h of discharge or referral.
2. Rapid follow-up: SOS navigators were to send handwritten notes (i.e., caring contact cards) within seven (7) days of discharge from ED or hospitalization or referral from another agency.
3. Care transition: SOS navigators aimed to connect enrollees to mental health providers, insurance navigators, and community support agencies as needed.
4. Safety plans: If safety plans were not on record, SOS navigators assessed the client’s access to lethal means and also developed a safety plan.

In sum, the support provided by the SOS team was acute crisis response, designed to provide caring contact, rapid follow-up, care transition protocols for patient safety, and connection to community recovery agencies. SOS navigators were also available via text message and phone to provide just-in-time support for individuals. The overarching goal was for SOS navigators to walk alongside patients in their care for 12 months of the program or to discharge individuals who no longer needed assistance.

### 2.2. Participants

Over the course of one year, there were 1909 referrals to the program and 408 patients enrolled. These enrollees worked with their SOS navigator for at-elbow support for suicide, domestic violence, or other mental health treatment. These 408 patients had an average age of 40.0 years (SD = 11) and were 51.5% female. About one-fourth of the enrollees (*n* = 108) screened positive for IPV at intake. Although care and support were provided to all enrollees, this paper focuses only on those individuals who screened positive for IPV.

### 2.3. Measures

#### 2.3.1. Partner Violence

All enrollees completed a brief 3-item partner violence screen [20] designed to be delivered in emergency departments and focused on partner violence in the past year and feelings of safety with current and past partner(s). The PVS contained the following items: “Have you been hit, kicked, punched, or otherwise hurt by someone within the past year? If so, by whom?”; “Do you feel safe in your current relationship?”; “Is there a partner from a previous relationship who is making you feel unsafe now?” If a person indicated they had experienced violence or did not feel safe due to a current or past partner, they screened positive for IPV and were directed to appropriate resources.

#### 2.3.2. Suicidality

Enrollees completed the Columbia–Suicide Severity Rating Scale (C-SSRS), a validated instrument used to quantify the severity of suicidal ideation and behavior [21]. The C-SSRS has been deemed suitable for use in clinical settings, public settings, and even everyday situations as it is brief and includes screening for a wide range of risk factors. Questions included the following (pertaining to the past month): “1. Have you wished you were dead or wished you could go to sleep and not wake up?” and “2. Have you actually had any thoughts about killing yourself?” If respondents answered ‘yes’ to having thoughts about suicide, then the following questions were asked: “3. Have you been thinking about how you might do this?”; “4. Have you had these thoughts and had some intention of acting on them?”; “5. Have you started to work out or worked out the details of how to kill yourself? Did you intend to carry out this plan?” Finally, all respondents were asked “6. Have you done anything, started to do anything, or prepared to do anything to end your life.” A “yes” response to Questions 3 or 6 (not within the past 3 months) was categorized as moderate risk; a “yes” response to 4, 5, or 6 (within the past 3 months) was categorized as high risk. Low risk represented a low level of suicidal ideation without a plan or intent; whereas high risk indicated active suicidal ideation with a specific plan and intent, and moderate was in between. Based on their classification and clinical decision-making, patients were referred to a relevant program for a standardized evidence-based pathway of treatment.

#### 2.3.3. Depression

Enrollees completed the 9-item Patient Health Questionnaire (PHQ-9), a validated instrument used to quantify the severity of depression symptoms [22]. Individuals indicated how often in the last two weeks they were bothered by a variety of problems using a 4-point Likert scale (0 = not at all, 3 = nearly every day), with a total maximum score of 27. Items included the following: “little interest or pleasure in doing things”, “feeling down, depressed, or hopeless”, “feeling tired or having little energy”, and “trouble concentrating on things, such as reading the newspaper or watching television”. Scores of 5–9 were categorized as mild depression, 10–14 as moderate, 15–19 as moderately severe, and 20–27 as severe. Based on the total score and clinical decision-making, patients were, if necessary, referred to a program for a standardized evidence-based pathway of treatment.

### 2.4. Statistical Analyses

Descriptive statistics were calculated for all measures. Using chi-square tests of independence and independent sample *t*-tests, the sample with suicidality was compared to the sample with IPV on age, gender, race, and depression (PHQ-9) scores at intake. To test for change in IPV and suicidality between intake and 6 months, a non-parametric paired test (McNemar) was used. Similarly, to test for change in depression, a paired samples *t*-test was conducted. Finally, an independent samples *t*-test was conducted for men and women using depression scores at intake and 6 months.

## 3. Results

### 3.1. Peer Support Program Aims

With regard to peers meeting the program aims with the enrollees, they were overall successful. In terms of crisis response, SOS navigators were able to enroll 58% of clients within 48 h of discharge or referral, and when this period was expanded to within seven (7) days of crisis, 75% of enrollees were reached within that timeframe. Additionally, rapid follow-up caring contact cards were sent to 91% of referred individuals within seven days of referral. In terms of care transition, 100% of enrollees were referred to mental health services, and 60% were referred to various community support agencies. Of the 6% of enrollees who had no form of health insurance, 100% were referred to insurance navigators as part of a standard process in our hospital system. Finally, SOS navigators created safety plans for all enrollees who did not have safety plans in their records.

### 3.2. Enrollee Characteristics at Intake

We first examined the baseline differences in enrollee characteristics between the 108 patients who screened positive for IPV (with or without suicidality) and the remaining 300 patients who screened positive for suicidality only. When compared to individuals with suicidality only, individuals who screened positive for IPV (with or without suicidality) at intake had similar ages (*t*(231) = 1.63, *p* = 0.10) and depression scores (*t*(406) = 0.99, *p* = 0.33). Additionally, there were no significant racial differences between these two groups in terms of their identifying as White (*X*^2^(1) = 0.91, *p* = 0.34) or Black (*X*^2^(1) = 0.02, *p* = 0.88). However, there were some gender differences. Specifically, a higher proportion of females screened positive for IPV (with or without suicidality) compared to suicidality only (*X*^2^(1) = 16.19, *p* < 0.001).

### 3.3. IPV Group Characteristics at Intake and Six Months

As Table 1 shows, over the course of six months, the original sample of 108 IPV patients was reduced to 63 participants (58.3% retention rate). Of the 45 individuals who did not complete the 6-month follow-up, 6 individuals agreed that they no longer needed service or refused to continue in the study; however, 38 individuals (84%) were unable to be reached and were administratively withdrawn from the program. One patient died prior to the six-month follow-up. Demographic characteristics of the retained sample at intake and 6 months are shown in Table 1.

### 3.4. Mental Health Statistics of IPV Group over 6 Months

As shown in Table 2, the group of individuals who were retained in the program for six months reported lower depression scores than the original IPV cohort. Additionally, fewer reported partner violence and fewer were screened at moderate or high levels of risk for suicide. However, as these differences could be attributed to sample differences, we conducted additional analyses with only the subsample of individuals who completed both assessments.

As shown in Table 3, depression, partner violence, and suicidality were significantly reduced in this subsample over the course of their six-month participation in the program. These differences could be due to the effectiveness of the program or the fact that those who had fewer mental health symptoms were more likely to maintain their enrollment (i.e., attrition issues leading to a biased sample).

### 3.5. Gender Differences in Depression in IPV Group over 6 Months

Finally, as shown in Table 4, though men reported more depression symptoms, there was not a statistically significant difference in depression symptoms between men and women at intake (stat) or at 6 months (stat).

## 4. Discussion

Previously, peer support programs have proven effective for suicide prevention [23] and depression [24], and peer support was deemed important by patients who utilize mental health services [2]. More recently, peer support programs have been shown to be helpful for those who have experienced IPV, and one of the greatest strengths of these programs is assisting an individual with building social capital in various forms, including information, competencies, connections to social networks, and reduction in stigma [25]. Our pilot peer support program, focused on continuity of care and recovery support for patients with multimorbidity involving mental health-related issues, had the aim of providing similar opportunities for building social capital. Specifically, we focused on providing at-elbow, wraparound support for women and men who experienced domestic violence, most of whom also had a moderate to high risk of suicidality and depression symptoms.

Overall, we were able to attract a large number of referrals (*n* = 1909) to the program, which is likely attributable to our health system’s strong community connections. During the entire course of the grant, we had support for the program from acute inpatient care, emergency care, and inpatient and outpatient behavioral health services. We also built meaningful connections with primary care providers and community agencies that offered a variety of wraparound services. This support and connection allowed us to enroll and provide services to 108 individuals who reported IPV. Building these connections within the community through our previous peer support program (focused on OUD/SUD recovery) allowed us to easily and quickly access resources with few structural barriers, and we were able to connect all enrollees with mental health services and many of them (60%) with community organizations to meet housing, transportation, and other support needs. To facilitate the coordinated care effort needed to address the multimorbidity of mental health issues, we recommend that health systems or other service organizations begin to build these community-based relationships prior to the implementation of a peer support program.

Research has shown that IPV victims who have attempted suicide are more likely to access DV services than those who do not have a suicide attempt [13]. Although this previous research has focused primarily on women, in our sample that included both men and women, the largest proportion of participants at intake screened positive for IPV and had at least a moderate risk of suicidality. This may be a sampling bias due to the SOS navigators being associated with EDs; however, our data provide some support for these previous findings [13] in terms of the utilization of peer support. In other words, those coping with multiple challenges that may affect mental health may be the ones most likely to make use of peer support services. This is an encouraging finding, as those who need the most help may be getting the most help, and it provides a model for healthcare and DV agencies to address individuals with multiple mental health needs.

Just over half of the enrollees (58%) stayed enrolled for six months. During that time, they received regular phone calls and text messages from the SOS navigators, who assisted them with accessing resources applicable to their ongoing needs, which included anything from transportation to connections with outpatient or inpatient psychiatric services. The individuals who stayed in the program did not differ from the initial cohort in terms of demographics or intake category; however, those still enrolled after six months had significantly lower depression scores, were less likely to still be experiencing IPV, and were less likely to be suicidal compared to their intake. This finding suggests the program may have been effective, or it could also be that those who stay enrolled in programs long-term had a natural tendency to see long-term positive results. In either case, a structured program to provide at-elbow assistance may be helpful to many of these multimorbid patients who need continuity of care.

Finally, it is notable that men made up more than one-fourth of this cohort of multimorbid individuals reporting IPV victimization, suicidality, and depression. These findings provide support for research involving men showing links between IPV victimization and suicidality [11,16] but are in contrast to the studies that found no link among men between suicidality and IPV victimization [14,15] and an inverse relationship between IPV and depression [15]. Perhaps the recent suicide attempt that triggered the SOS team involvement for many participants was a sample characteristic that accounts for these findings. Alternatively, perhaps there is a time effect, and more studies provide a more accurate depiction of the state of men’s mental health and IPV experiences than the decade-old review. Future research should explore how gender and multimorbidity influence the relationship between IPV and suicidality.

### Limitations

This study does have limitations that need mentioning. First, this was a pilot project, and it did not include random assignment nor a control group similar to the reference sample that received no peer support. Thus, although individuals showed improvement over the course of the program, this could be due to time or other factors. Randomized, control studies examining peer navigation with these populations are necessary to provide more support for the program. Additionally, the individuals in this program were self-selected to participate after referral. Previous research has shown that individuals who take part in interventions may have fewer health disparities than those who do not participate in interventions [26]. This creates a selection bias towards healthy volunteers, and consequently, the results may not be generalizable to all populations of individuals who are coping with DV and particularly those who may have the greatest physical and/or mental health needs. In addition to these recruitment issues, we faced a high rate of attrition in our study, and our final sample was small compared to the original cohort. As attrition in health-related studies is not typically random, and it is likely that those with the most challenges have the lowest levels of engagement [27], our final sample may not be representative of the original sample. Our results need to be interpreted with these limitations in mind, and future research should aim to expand upon these preliminary findings with similar samples of multimorbid individuals.

## 5. Conclusions

Overall, we were encouraged by the outcomes of our pilot program in providing support for hundreds of individuals facing a number of mental health and environmental challenges. Multimorbidity of mental health needs can be addressed through peer support, and interconnected webs of community support are essential to meet the varied needs of those experiencing DV and suicidality.

## Figures and Tables

**Table 1:** Sample Characteristics.

	Intake (*n* = 108)	6 Month (*n* = 63)
Age (Years)		
Mean (SD)	38.54 (9.90)	39.24 (10.01)
Gender, *n* (%)		
Women	73 (67.6%)	45 (71.4%)
Men	31 (28.7%)	16 (25.4%)
Transgender	2 (1.9%)	1 (1.6%)
Other	2 (1.9%)	1 (1.6%)
Hispanic, *n* (%)		
No	100 (92.6%)	58 (92.1%)
Yes	6 (5.6%)	4 (6.3%)
Missing	2 (1.9%)	1 (1.6%)
Race, *n* (%)		
Alaska Native	1 (0.9%)	0 (0.0%)
American Indian	17 (15.7%)	10 (15.9%)
Asian	2 (1.9%)	1 (1.6%)
Black	18 (16.7%)	7 (11.1%)
Native Hawaiian	1 (0.9%)	0 (00.0%)
White	90 (83.3%)	53 (84.1%)
Missing	2 (1.9%)	1 (1.6%)
Insurance, *n* (%)		
Commercial/Third Party	22 (20.4%)	14 (22.2%)
Medicaid	70 (64.8%)	41 (65.1%)
Medicare/Medicaid	4 (3.7%)	0 (0.0%)
Medicare/Medicare Replacement	7 (6.5%)	6 (9.5%)
VA/Tricare	2 (1.9%)	2 (3.2%)
None	3 (2.8%)	0 (0.0%)

Note: Multiple Race categories could be selected.

**Table 2:** Summary statistics for Depression, Partner Violence, and Suicidality by Time.

	Time	
Measure	Intake (*n* = 108)	6 Month (*n* = 63)
Depression		
Mean (SD)	17.8 (6.6)	10.3 (7.7)
Partner Violence, *n* (%)		
No	0 (0.0%)	39 (61.9%)
Yes	108 (100.0%)	24 (38.1%)
Suicidality, *n* (%)		
Low Risk	38 (35.2%)	29 (46.0%)
Moderate Risk	22 (20.4%)	26 (41.3%)
High Risk	48 (44.4%)	8 (12.7%)

**Table 3:** Summary statistics for Depression, Partner Violence, and Suicidality by Time for Enrollees Completing Both Intake and 6-month Assessments.

	Time		Test Statistic
Measure	Intake (*n* = 63)	6 Month (*n* = 63)	
Depression			
Mean (SD)	17.9 (6.7)	10.3 (7.7)	*t*(62) = 8.20, *p* < 0.001
Partner Violence, *n* (%)			
No	0 (0.0%)	39 (61.9%)	*X*^2^(1) = 37.03,
Yes	63 (100.0%)	24 (38.1%)	*p* < 0.001
Suicidality, *n* (%)			
Low Risk	20 (31.8%)	29 (46.0%)	*X*^2^(3) = 22.78,
Moderate Risk	13 (20.6%)	26 (41.3%)	*p* < 0.001
High Risk	30 (47.6%)	8 (12.7%)	

**Table 4:** Men’s and Women’s Depression Scores at Intake and 6 months.

Gender	Depression Mean (SD)	
	Intake	6 Month
Men	18.39 (5.91)	12.56 (8.02)
Women	17.22 (6.77)	9.71 (7.62)

Note: At intake, *n* = 31 men and *n* = 73 women; at 6 months, *n* = 16 men and *n* = 45 women with PHQ-9 scores.

## Data Availability

Due to patient confidentiality and the potential to cross-reference multiple risk factors of individuals in this region, no data from this study will be shared.
